# Impact of fetal and maternal characteristics on the accuracy and precision of sonographic fetal weight estimation: population‐based study

**DOI:** 10.1002/uog.70117

**Published:** 2025-10-24

**Authors:** M. Granfors, Ä. Mantel, O. Axelsson, S. Cnattingius, L. Lindström

**Affiliations:** ^1^ Division of Clinical Epidemiology, Department of Medicine (Solna) Karolinska Institutet Stockholm Sweden; ^2^ Department of Women's Health, Division of Obstetrics Karolinska University Hospital Stockholm Sweden; ^3^ Department of Women's and Children's Health, Uppsala University Uppsala Sweden; ^4^ Centre for Clinical Research Sörmland Uppsala University Eskilstuna Sweden

**Keywords:** accuracy, estimated fetal size, fetal presentation, fetal sex, fetal weight estimation, large‐for‐gestational age, precision, small‐for‐gestational age, ultrasound

## Abstract

**Objective:**

Ultrasound‐based fetal growth assessment is an essential part of clinical decision‐making in modern obstetric care. This study aimed to evaluate the impact of maternal and fetal characteristics on the accuracy and precision of sonographic fetal weight estimation.

**Methods:**

We included 31 521 singleton pregnancies registered in the Swedish Pregnancy Register between January 2014 and December 2021, with delivery ≥ 22 + 0 weeks' gestation and estimated fetal weight (EFW) assessed using ultrasound within 2 days before delivery. Fetal biometric ultrasound measurements were used to calculate EFW according to the formula of Persson and Weldner. Mean percentage error (MPE) and the proportion of EFW within ± 10% of the birth weight were calculated and stratified by maternal body mass index (BMI), fetal sex, fetal presentation at birth, gestational age (GA) at ultrasound and standardized EFW (estimated small‐for‐gestational age (SGA), appropriate‐for‐gestational age (AGA) or large‐for‐gestational age). Univariable and adjusted risk ratios (aRRs) for poor EFW assessment, defined as EFW deviating more than ± 10% from the birth weight, were calculated.

**Results:**

All investigated fetal characteristics, but not maternal BMI, affected the precision of EFW. The inaccuracies of fetal weight estimation were most pronounced in fetuses estimated as severe SGA (< 3^rd^ percentile) across all subgroups of GA at ultrasound (overall MPE, −5.4 ± 9.8%; 67% of estimates within ± 10% of birth weight). The risk of poor fetal weight estimation was highest for severe‐SGA‐estimated *vs* AGA‐estimated fetuses (aRR, 1.44 (95% CI, 1.38–1.51)). Additionally, the risk of poor fetal weight estimation was higher for male *vs* female fetuses (aRR, 1.09 (95% CI, 1.05–1.14)), non‐cephalic *vs* cephalic presentation (aRR, 1.20 (95% CI, 1.13–1.28)) and preterm *vs* late term (39 + 0 to 40 + 6 weeks) GA at ultrasound, with the highest risk observed for those with GA of 22 + 0 to 27 + 6 weeks at ultrasound (aRR, 1.30 (95% CI, 1.13–1.50)).

**Conclusions:**

All assessed fetal characteristics, especially standardized EFW, affected the accuracy of ultrasonographic fetal weight estimation. Understanding systematic errors in the EFW formula used is of utmost importance in clinical decision‐making to prevent unnecessary or missed obstetric interventions. © 2025 The Author(s). *Ultrasound in Obstetrics & Gynecology* published by John Wiley & Sons Ltd on behalf of International Society of Ultrasound in Obstetrics and Gynecology.

## INTRODUCTION

Ultrasound examination for fetal weight estimation is a routine practice performed daily in clinical settings worldwide, with critical clinical decisions often guided by these weight estimations. It has long been known that small‐for‐gestational‐age (SGA) infants at any gestational age (GA) suffer the highest morbidity and mortality rates^1^. Fetuses with fetal growth restriction (FGR) are particularly vulnerable to adverse perinatal outcomes, and unrecognized FGR is the single largest risk factor for stillbirth^2^, ^3^, ^4^. Moreover, large‐for‐gestational‐age (LGA) infants are also predisposed to a range of adverse perinatal outcomes, and fetal macrosomia is a significant risk factor for maternal delivery complications^5^, ^6^. While accurate identification of deviant fetal growth can improve outcomes, inaccuracies in fetal weight estimation can increase the risk of both absent and unnecessary interventions, potentially compromising perinatal and maternal outcomes^3^, ^7^, ^8^.

To assess the accuracy of fetal weight estimation, the estimated fetal weight (EFW) is usually compared with the actual birth weight (BW), with a recommended scan‐to‐delivery interval of no more than 7 days^9^, ^10^. Despite numerous efforts to develop new and improved models for EFW, the formula of Hadlock *et al*. published in 1985, based on head circumference, abdominal circumference and femur length measurements, remains the most accurate for predicting BW^10^, ^11^, ^12^. However, in Sweden, the EFW formula of Persson and Weldner^13^ published in 1986, which includes biparietal diameter but not head circumference, is used. In a recent study, we found the formula of Persson and Weldner to be as good as or superior to other biparietal diameter‐based formulae^14^.

Unfortunately, EFW formulae tend to have the least accurate performance in cases in which precision is the most critical, namely in fetuses with low or high EFW or those born extremely preterm or post‐term^14^, ^15^, ^16^. Other factors associated with EFW performance have been investigated separately in smaller studies, yielding inconsistent results^16^, ^17^, ^18^, ^19^. To our knowledge, no large‐scale study has examined the accuracy of fetal weight estimation across gestation, taking into account standardized EFW and other factors simultaneously.

This study aimed to assess the impact of maternal and fetal characteristics on the accuracy and precision of sonographic fetal weight estimation, utilizing population‐based, prospectively collected data.

## METHODS

### Data sources and study population

This prospective cohort study comprised 31 521 singleton pregnancies registered in the Swedish Pregnancy Register (SPR) between 1 January 2014 and 31 December 2021, with delivery ≥ 22 + 0 weeks' gestation and EFW assessed using ultrasound within 2 days before delivery. During the study period, routine second‐trimester ultrasound screening at around 18–19 weeks was offered to all pregnant women in Sweden, in accordance with the national standard of prenatal care. Subsequent ultrasonographic estimations of fetal weight were performed only when clinically indicated, such as in cases of maternal hypertension, pre‐eclampsia, diabetes mellitus, advanced maternal age, conception via *in‐vitro* fertilization, abnormal symphysis–fundus measurement or other relevant indications. Admission to the delivery ward was not, in itself, an indication for fetal weight estimation. Consequently, only a minority of pregnant women in Sweden had an ultrasound‐based EFW assessment within 2 days before delivery.

The SPR contains detailed, prospectively collected data on pregnancy, delivery and the postpartum period in Sweden^20^. Stillbirths and cases diagnosed with structural anomaly or chromosomal aberration (defined as International Classification of Diseases codes Q00–Q99) were not included in the study population. Detailed descriptions of the SPR, the study population and a Swedish reference population, which includes all women with a singleton liveborn neonate delivered ≥ 22 + 0 weeks registered in the SPR during the study period, irrespective of whether EFW was assessed within 2 days before delivery (*n* = 804 915), are available in previous publications^14^, ^20^.

Fetal biometric ultrasound measurements were used to calculate EFW according to the formula of Persson and Weldner^13^.

### Fetal and maternal characteristics

The cohort was stratified by maternal body mass index (BMI) (< 25.0 kg/m^2^, 25.0–29.9 kg/m^2^ or ≥ 30.0 kg/m^2^), fetal sex (female or male), fetal presentation at birth (cephalic or non‐cephalic), GA at ultrasound (22 + 0 to 27 + 6 weeks, 28 + 0 to 31 + 6 weeks, 32 + 0 to 36 + 6 weeks, 37 + 0 to 38 + 6 weeks, 39 + 0 to 40 + 6 weeks or ≥ 41 + 0 weeks) and standardized EFW. Each measurement of EFW was standardized for GA, classified in percentiles according to the new Swedish sex‐neutral intrauterine growth reference ranges^21^, defined as: severe SGA (EFW < 3^rd^ percentile); mild SGA (EFW 3^rd^ to < 10^th^ percentile); appropriate‐for‐gestational age (AGA) (EFW 10^th^–90^th^ percentile); mild LGA (EFW > 90^th^ to 97^th^ percentile); or severe LGA (EFW > 97^th^ percentile).

### Statistical analysis

First, the accuracy and predictive performance of EFW were evaluated for each subgroup using the following metrics. (1) Mean percentage error (MPE), which also represents the systematic error, was calculated as the mean of the percentage errors using the formula: ((EFW − BW)/EFW) × 100. Using EFW as the denominator, instead of BW, aligns with the information most relevant to clinicians for prebirth decisions^22^. One‐way ANOVA was used to compare MPEs across subgroups, with *post*‐*hoc* testing using Bonferroni correction for multiple comparisons. (2) Random error, which represents the non‐systematic part of the prediction error, calculated as the SD of the MPE. (3) Proportion of accurate estimates, defined as EFW within ± 10% of the actual BW. The chi‐square test was used to estimate the difference between groups in accuracy in weight estimation.

Second, we calculated the relative risk of poor fetal weight estimation, defined as EFW deviating more than ± 10% from the BW, for all factors that showed a statistically significant association with estimation accuracy. The relative risk was expressed as univariable and adjusted risk ratios (RRs) with 95% CIs and was calculated using log‐binomial multivariable logistic regression models, with male fetal sex, cephalic presentation, GA at ultrasound 39 + 0 to 40 + 6 weeks and estimated AGA, respectively, as reference groups. Adjustments were then made for fetal sex (female/male), fetal presentation at birth (cephalic/non‐cephalic), GA at ultrasound (continuous variable) and standardized EFW (continuous variable), when applicable. Only cases with complete data were included in the adjusted models.

Third, to further examine the robustness of the results, we reported stratum‐specific estimates for each subgroup (fetal sex, fetal presentation at birth and GA at ultrasound). To assess potential effect modification by subgroup, an interaction term between the predictive variable (standardized EFW) and potential modifier (fetal sex, fetal presentation at birth and GA at ultrasound, respectively) was included in the regression model for the factor with the strongest association with poor fetal weight estimation, with statistical significance set at *P* < 0.05.

Fourth, MPE ± SD and the proportion of EFW within ± 10% of BW were stratified by fetal sex, fetal presentation at birth and GA at ultrasound, respectively, all by standardized EFW at ultrasound. Finally, univariable RRs and adjusted RRs (aRRs) for poor fetal weight estimation were stratified by, and calculated for, the two factors with the strongest association with poor fetal weight estimation.

Statistical analysis was performed using IBM SPSS Statistics for Windows version 25.0 (IBM Corp., Armonk, NY, USA). The study was approved by the National Ethical Review Board (Diary number 2021‐03123). All procedures involving humans were carried out according to the ethical standards of the 1964 Helsinki Declaration. All registry data were merged and pseudonymized before delivery to the research group; therefore, informed consent was not required.

## RESULTS

Overall, 31 521 singleton pregnancies were included in the study population. Maternal and neonatal characteristics of the study population and the Swedish reference population (*n* = 804 915) have been reported previously^14^ and are presented in Table S1. Notable differences were observed between the study population and the reference population, particularly in the proportions of preterm birth < 37 weeks (15.5% *vs* 4.2%) and severe SGA at birth (24.8% *vs* 9.0%).

Table 1 shows the accuracy and precision of sonographic fetal weight estimation, stratified by maternal BMI, fetal sex, fetal presentation at birth, GA at ultrasound and standardized EFW. There were significant differences in the precision of fetal weight estimation for all fetal characteristics, but not for maternal BMI. Fetal weight estimation was more accurate in female compared with male fetuses (76.1*% vs* 73.6% of estimates within ± 10% of BW, respectively), cephalic compared with non‐cephalic presentation (72.8% *vs* 67.8%, respectively) and when ultrasound was performed at a later GA (gradually increasing proportion of estimates within ± 10% of BW from 65.5% for GA at ultrasound < 28 + 0 weeks to 76.5% for GA at ultrasound ≥ 41 + 0 weeks). Differences in precision were most pronounced when the cohort was stratified by standardized EFW, with the lowest accuracy observed in fetuses estimated as severe SGA (MPE, –5.4 ± 9.8%; 67.0% of estimates within ± 10% of BW) and the highest in fetuses estimated as mild LGA (MPE, 1.6 ± 7.3%; 82.6% of estimates within ± 10% of BW). BW was underestimated in 69.9% of fetuses estimated as severe SGA and 39.0% of fetuses estimated as severe LGA (*P* < 0.001).

**Table 1:** Accuracy and precision of sonographic weight estimation stratified by maternal body mass index (BMI), fetal sex, fetal presentation at birth, gestational age at ultrasound and standardized estimated fetal weight (EFW) in study population (*n* = 31 521)

Variable	Prevalence	MPE ± SD (%)	*P* *	EFW within ± 10% of BW	*P* †
Maternal BMI‡			0.503		0.078
< 25.0 kg/m^2^	15 723/29 695 (52.9)	–2.6 ± 8.7		11 882 (75.6)	
25.0–29.9 kg/m^2^	8103/29 695 (27.3)	–2.6 ± 9.1		6025 (74.4)	
≥ 30.0 kg/m^2^	5869/29 695 (19.8)	–2.5 ± 9.0		4376 (74.6)	
Fetal sex§			< 0.001		< 0.001
Female	15 397/31 519 (48.8)	–2.1 ± 8.9		11 712 (76.1)	
Male	16 122/31 519 (51.2)	–3.3 ± 9.0		11 870 (73.6)	
Fetal presentation at birth			< 0.001		< 0.001
Cephalic	28 911 (91.7)	–2.6 ± 8.8		21 046 (72.8)	
Non‐cephalic	2610 (8.3)	–4.4 ± 10.1		1770 (67.8)	
Gestational age at ultrasound			< 0.001		< 0.001
22 + 0 to 27 + 6 weeks	412 (1.3)	–4.3 ± 11.3		270 (65.5)	
28 + 0 to 31 + 6 weeks	789 (2.5)	–2.9 ± 10.5		534 (67.7)	
32 + 0 to 36 + 6 weeks	4073 (12.9)	−4.1 ± 9.5		2839 (69.7)	
37 + 0 to 38 + 6 weeks	7408 (23.5)	−3.1 ± 8.8		5570 (75.2)	
39 + 0 to 40 + 6 weeks	11 568 (36.7)	−2.4 ± 8.7		8795 (76.0)	
≥ 41 + 0 weeks	7271 (23.1)	−1.9 ± 8.8		5565 (76.5)	
Standardized EFW			< 0.001		< 0.001
Severe SGA (< 3^rd^ percentile)	8381 (26.6)	–5.4 ± 9.8		5614 (67.0)	
Mild SGA (3^rd^ to < 10^th^ percentile)	4540 (14.4)	–3.7 ± 8.6		3356 (73.9)	
AGA (10^th^–90^th^ percentile)	15 402 (48.9)	–1.8 ± 8.3		12 030 (78.1)	
Mild LGA (> 90^th^ to 97^th^ percentile)	1651 (5.2)	1.6 ± 7.3		1363 (82.6)	
Severe LGA (> 97^th^ percentile)	1547 (4.9)	1.6 ± 8.0		1220 (78.9)	

Data are given as *n*/*N* (%) or *n* (%), unless stated otherwise.

* One‐way ANOVA with *post‐hoc* Bonferroni correction testing for multiple comparisons.

† Chi‐square test.

‡ Data missing for 1826 cases.

§ Data missing for two cases. AGA, appropriate‐for‐gestational age; BW, birth weight; LGA, large‐for‐gestational age; MPE, mean percentage error; SGA, small‐for‐gestational age.

### Risk of poor fetal weight estimation by fetal characteristics

Univariable and adjusted RRs for poor fetal weight estimation by fetal sex, fetal presentation at birth, GA at ultrasound and standardized EFW, respectively, are presented in Table 2. All fetal characteristics showed significant variation across most subgroups. The risk of poor fetal weight estimation was higher in male *vs* female fetuses (aRR, 1.09 (95% CI, 1.05–1.14)); non‐cephalic *vs* cephalic presentation (aRR, 1.20 (95% CI, 1.13–1.28)); preterm *vs* late term (39 + 0 to 40 + 6 weeks) GA at ultrasound (22 + 0 to 27 + 6 weeks: aRR, 1.30 (95% CI, 1.13–1.50); 28 + 0 to 31 + 6 weeks: aRR, 1.26 (95% CI, 1.13–1.40); and 32 + 0 to 36 + 6 weeks: aRR, 1.17 (95% CI, 1.10–1.24)); and SGA‐estimated *vs* AGA‐estimated fetuses (severe SGA: aRR, 1.44 (95% CI, 1.38–1.51); mild SGA: aRR, 1.17 (95% CI, 1.10–1.24)). Moreover, mild LGA was associated with a trend towards a decreased risk of poor fetal weight estimation in comparison with AGA (aRR, 0.90 (95% CI, 0.81–1.00)).

**Table 2:** Univariable and adjusted risk ratios (RRs) for poor fetal weight estimation stratified by fetal sex, fetal presentation at birth, gestational age at ultrasound and standardized estimated fetal weight (EFW)

Variable	Univariable RR (95% CI)	Adjusted RR (95% CI)
Fetal sex		
Female	1 (reference)	1 (reference)
Male	1.06 (1.02–1.10)	1.09 (1.05–1.14)
Fetal presentation at birth		
Cephalic	1 (reference)	1 (reference)
Non‐cephalic	1.30 (1.22–1.38)	1.20 (1.13–1.28)
Gestational age at ultrasound		
22 + 0 to 27 + 6 weeks	1.47 (1.28–1.69)	1.30 (1.13–1.50)
28 + 0 to 31 + 6 weeks	1.40 (1.26–1.56)	1.26 (1.13–1.40)
32 + 0 to 36 + 6 weeks	1.23 (1.16–1.31)	1.17 (1.10–1.24)
37 + 0 to 38 + 6 weeks	1.04 (0.98–1.09)	1.05 (0.99–1.10)
39 + 0 to 40 + 6 weeks	1 (reference)	1 (reference)
≥ 41 + 0 weeks	1.01 (0.96–1.07)	1.00 (0.95–1.06)
Standardized EFW		
Severe SGA (< 3^rd^ percentile)	1.46 (1.40–1.53)	1.44 (1.38–1.51)
Mild SGA (3^rd^ to < 10^th^ percentile)	1.16 (1.09–1.23)	1.17 (1.10–1.24)
AGA (10^th^–90^th^ percentile)	1 (reference)	1 (reference)
Mild LGA (> 90^th^ to 97^th^ percentile)	0.90 (0.81–1.00)	0.90 (0.81–1.00)
Severe LGA (> 97^th^ percentile)	1.11 (1.01–1.22)	1.10 (0.996–1.21)

All estimates are adjusted for relevant combinations of the following variables, depending on the specific analysis: fetal sex (male/female), fetal presentation at birth (cephalic/non‐cephalic), gestational age at ultrasound (continuous variable) and standardized EFW (continuous variable). Poor fetal weight estimation defined as EFW deviating by more than ± 10% from birth weight. AGA, appropriate‐for‐gestational age; LGA, large‐for‐gestational age; SGA, small‐for‐gestational age.

### Multiplicative interaction for poor fetal weight estimation by standardized EFW

Since standardized EFW had the greatest impact on the risk of poor fetal weight estimation, we tested for multiplicative interactions. Significant interaction terms were found between standardized EFW and fetal sex (*P* = 0.027) and between standardized EFW and fetal presentation at birth (*P* = 0.013). There was no significant interaction between standardized EFW and GA (*P* = 0.477). Univariable RRs and aRRs for poor fetal weight estimation in stratified regression analyses by fetal sex and fetal presentation at birth are shown in Tables S2 and S3, respectively. Compared with fetuses classified as AGA, the risk of poor fetal weight estimation was highest in non‐cephalic fetuses classified as severe SGA (aRR, 1.74 (95% CI, 1.52–2.00)).

### Risk of poor fetal weight estimation by GA and standardized EFW

Table 3 shows univariable RRs and aRRs for poor fetal weight estimation by GA at ultrasound and standardized EFW, using GA ≥ 37 weeks at ultrasound and standardized EFW between the 3^rd^ and 97^th^ percentiles as the reference. Irrespective of GA at ultrasound, the risk of poor fetal weight estimation was highest in fetuses assessed as severe SGA, the risk being most pronounced in extreme preterm and very preterm fetuses (GA at ultrasound < 32 weeks). Preterm fetuses generally had a higher risk of poor fetal weight estimation, regardless of standardized EFW.

**Table 3:** Univariable and adjusted risk ratios (RRs) for poor fetal weight estimation by gestational age at ultrasound and standardized estimated fetal weight (EFW)

Variable	Univariable RR (95% CI)	Adjusted RR* (95% CI)
22 + 0 to 31 + 6 weeks		
Severe SGA	1.74 (1.56–1.95)	1.63 (1.46–1.83)
Mild SGA, AGA, mild LGA	1.38 (1.21–1.57)	1.31 (1.15–1.49)
Severe LGA	1.33 (0.63–2.80)	1.29 (0.62–2.69)
32 + 0 to 36 + 6 weeks		
Severe SGA	1.57 (1.45–1.69)	1.52 (1.41–1.65)
Mild SGA, AGA, mild LGA	1.20 (1.11–1.29)	1.17 (1.09–1.26)
Severe LGA	1.42 (1.18–1.72)	1.41 (1.17–1.71)
≥ 37 + 0 weeks		
Severe SGA	1.43 (1.36–14.9)	1.44 (1.37–1.51)
Mild SGA, AGA, mild LGA	1 (reference)	1 (reference)
Severe LGA	1.05 (0.94–1.17)	1.04 (0.94–1.16)

* Adjusted for fetal sex and fetal presentation at birth. Poor fetal weight estimation defined as EFW deviating by more than ± 10% from birth weight. AGA, appropriate‐for‐gestational age; LGA, large‐for‐gestational age; SGA, small‐for‐gestational age.

### Stratified and subgroup analyses

**Figure 1:**
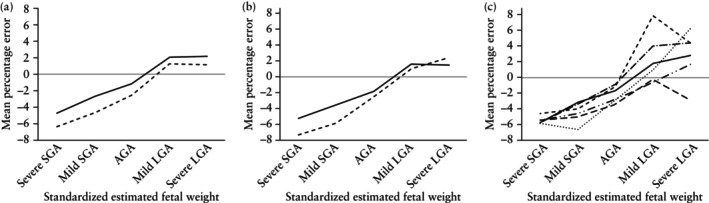
Precision of fetal weight estimation, stratified by fetal characteristics, according to standardized estimated fetal weight. (a) Stratified by fetal sex (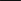, female; 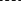, male). (b) Stratified by fetal presentation (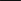, cephalic; 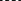, non‐cephalic). (c) Stratified by gestational age at ultrasound (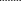, 22–27 weeks; 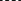, 28–31 weeks; 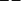, 32–36 weeks; 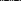, 37–38 weeks; 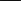, 39–40 weeks; 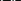, ≥ 41 weeks). AGA, appropriate‐for‐gestational age; LGA, large‐for‐gestational age; SGA, small‐for‐gestational age.

Stratified analyses for fetal sex, fetal presentation at birth and GA at ultrasound, all by standardized EFW, revealed similar patterns across all subgroups. Fetal weight estimation was the least accurate in fetuses assessed as severe SGA in all subgroups (fetal sex, fetal presentation at birth and GA at ultrasound), and generally the most accurate in fetuses estimated as mild LGA (Figure 1, Tables S4–S6). Furthermore, in all subgroups, there was a clear and consistent trend to a general underestimation of BW in fetuses estimated as severe SGA, and a general overestimation of BW in fetuses estimated as severe LGA.

## DISCUSSION

In this study of 31 521 singleton pregnancies with prospectively collected data on ultrasound‐based fetal weight estimation performed within 2 days before birth, standardized EFW had the greatest impact on the accuracy of fetal weight estimation. Fetuses estimated to be severe SGA had a high risk of poor fetal weight estimation, which was even more pronounced for those with lower GA at ultrasound. There was a clear and consistent trend of a general underestimation of BW in fetuses classified as severe SGA and a slight overestimation of BW in fetuses classified as severe LGA. Fetal sex and fetal presentation at birth showed significant effect modification in the risk of poor fetal weight estimation. Notably, male fetuses estimated as severe SGA, as well as fetuses estimated as severe or mild SGA in a non‐cephalic presentation, were associated with the highest risks for poor fetal weight estimation.

In many respects, our current results are in line with those of previous smaller studies. Larger inaccuracies in EFW are usually seen in SGA infants^17^, ^19^, ^23^, ^24^. Ben‐Haroush *et al*.^23^ found a risk of fetal weight underestimation in pregnancies with suspected FGR and overestimation in pregnancies with suspected LGA. Furthermore, several studies have investigated the impact of fetal sex and fetal presentation on the precision of fetal weight estimation, with significant findings in most, but not all, studies^16^, ^17^, ^25^. Less accurate fetal weight estimation is usually seen in fetuses with breech or non‐cephalic presentation^17^, ^25^. With few exceptions, and in line with our results, maternal BMI has been found to have no impact on the accuracy of EFW^16^, ^18^, ^19^. Partially in line with our results, two studies found limited or no impact of GA at ultrasound on the accuracy of EFW^18^, ^26^. Thus, our study demonstrates that, much like previously examined EFW formulae, the Persson and Weldner formula for EFW is the least accurate when applied in the smallest and most preterm fetuses.

The results regarding the performance of EFW in suspected macrosomia/LGA are inconsistent in the literature. While Scioscia *et al*.^27^ demonstrated a clear tendency among 35 different EFW formulae to underestimate fetal weight in those with a high BW, Zafman *et al*.^28^, consistent with our results, found that an EFW ≥ 4000 g was more likely to be an overestimation rather than an underestimation of actual BW.

Our study differs from previous research in several areas, which comprise key strengths of the study. First, our study population was, to our knowledge, markedly larger than any previous cohort in which the accuracy of fetal weight estimation has been investigated. Second, the SPR contains prospectively collected detailed data on pregnancy, delivery and the postpartum period, which precludes the risk of recall bias. Third, all fetal weight estimations were performed within 2 days before delivery, ensuring minimal error due to fetal growth between fetal weight estimation and birth. Most previous studies have applied a time interval of 3–7 days, and longer intervals may be strongly associated with estimation inaccuracy^18^. Fourth, MPE was determined using EFW as the denominator instead of BW, as clinical decisions are often made at the time of the ultrasound and thus are based on that information. Accordingly, this approach better reflects the clinical situation, which has been described previously^22^. To our knowledge, few previous studies have chosen this methodology. Fifth, all fetal and maternal characteristics investigated in this study, other than fetal presentation at birth, are potentially known at the time of fetal weight estimation, which makes the results directly translatable to clinical practice. Sixth, and most importantly, this study examined the association of several factors with EFW precision in the same study, thus reporting their mutual influence and potential interactions.

Our study has some limitations. First, and most notably, pregnancies included in this study were not comparable with the general pregnant population. Pregnancies at high risk of aberrant growth were included to a much greater extent, which may limit the generalizability of our results. Second, we did not have any detailed information regarding the competence of the ultrasound examiner. Third, we only had information on fetal presentation at birth and not at ultrasound, which may be considered a potential source of misclassification. Nevertheless, the probability that fetal presentation was the same on both occasions is high, since the interval between the ultrasound examination and birth was no longer than 2 days. Fourth, the observational study design might be considered a limitation, despite the inclusion of prospectively collected data from the SPR.

It is well known that the chosen EFW formula has a major impact on the precision and accuracy of fetal weight estimation. Data from our study and previous studies suggest that the patterns for many formulae might be comparable, with standardized EFW being the most important factor, having a significant impact on EFW accuracy, and with an additional impact of fetal sex, fetal presentation and GA at ultrasound. However, since some formulae tend to overestimate fetal weight, while others underestimate it, their accuracy may differ between fetuses assessed as SGA and those assessed as LGA. For example, EFW calculated using the Hadlock‐2 or Shepard's formula is most accurate in SGA‐estimated fetuses and least accurate in LGA‐estimated fetuses^14^. Moreover, it should be noted that the prevalence of SGA, AGA and LGA varies according to the definition and fetal size reference chart/standard used^29^.

It is essential to keep in mind the uncertainty and potential error of fetal weight estimation when basing clinical decisions on EFW. Knowledge of the weaknesses of the chosen formula, especially in the extremes of standardized EFW, is of great importance in clinical decision‐making. Hopefully, new technologies will render it possible to develop more accurate formulae. Until then, it is paramount in clinical decision‐making to be aware of inaccuracies in the chosen formula for fetal weight estimation.

In conclusion, this large‐scale study provides robust evidence that the accuracy and precision of fetal weight estimation are strongly influenced by standardized EFW at ultrasound. Fetuses that are estimated to be severe SGA showed the least accurate weight estimations, which was even more pronounced in the case of early GA at ultrasound. There was a clear and consistent trend of increasing underestimation of BW with decreasing relative estimated fetal size. Fetal sex and presentation showed effect modification in the risk of poor weight estimation. Knowledge regarding these systematic errors in the chosen ultrasound‐based EFW formula is of utmost importance in clinical decision‐making.

## Supporting information


**Table S1** Maternal and infant characteristics.
**Tables S2 and S3** Univariable and adjusted risk ratios for poor fetal weight estimation (estimated fetal weight (EFW) deviating more than ± 10% from birth weight) by standardized EFW, stratified by fetal sex (Table S2) and fetal presentation at birth (Table S3).
**Table S4** Accuracy and precision of sonographic weight estimation stratified for fetal sex, by standardized estimated fetal weight, of pregnancies with known fetal sex (*n* = 31 519).
**Table S5** Accuracy and precision of sonographic weight estimation stratified by presentation at birth, by standardized estimated fetal weight at ultrasound, of pregnancies with known presentation at birth (*n* = 30 450).
**Table S6** Accuracy and precision of sonographic weight estimation stratified by gestational age at ultrasound, by standardized estimated fetal weight (*n* = 31 521).

## Data Availability

Sharing individual data would compromise ethical standards and legal requirements. Thus, we are not able to share individual data.
