# Supplementary material for: Impact of fetal and maternal characteristics on the accuracy and precision of sonographic fetal weight estimation: population‐based study
Source: Ultrasound Obstet Gynecol. 2025 Oct 24;66(6):747–53. doi: 10.1002/uog.70117 (PMC12671939; doi:10.1002/uog.70117)
Supplement: Supplementary file 1 — Table S1 Maternal and infant characteristics. Tables S2 and S3 Univariable and adjusted risk ratios for poor fetal weight estimation (estimated fetal weight (EFW) deviating more than ± 10% from birth weight) by standardized EFW, stratified by fetal sex (Table S2) and fetal presentation at birth (Table S3). Table S4 Accuracy and precision of sonographic weight estimation stratified for fetal sex, by standardized estimated fetal weight, of pregnancies with known fetal sex (n = 31 519). Table S5 Accuracy and precision of sonographic weight estimation stratified by presentation at birth, by standardized estimated fetal weight at ultrasound, of pregnancies with known presentation at birth (n = 30 450). Table S6 Accuracy and precision of sonographic weight estimation stratified by gestational age at ultrasound, by standardized estimated fetal weight (n = 31 521). [file UOG-66-747-s001.docx]

**Table S1. Maternal and infant characteristics**

|  | **Study population**  **(n=31 521)** | | **Reference population***  **(n=804 915)** | |
| --- | --- | --- | --- | --- |
|  | **N** | **Percent** | **N** | **Percent** |
| **Country of birth** |  |  |  |  |
| Nordic country | 20 213 | 64.1 | 536 127 | 66.6 |
| Remaining Europe | 1704 | 5.4 | 44 511 | 5.5 |
| Asia and Oceania | 3990 | 12.7 | 95 903 | 11.9 |
| Africa | 2038 | 6.5 | 41 446 | 5.1 |
| North and South America | 366 | 1.2 | 8832 | 1.1 |
| Missing | 3210 | 10.2 | 78 096 | 9.7 |
| **Level of education, years** |  |  |  |  |
| <10 | 2457 | 7.8 | 56 507 | 7.0 |
| 10-12 | 9949 | 31.6 | 254 610 | 31.6 |
| >12 | 13 281 | 42.1 | 355 749 | 44.2 |
| Missing | 5834 | 18.5 | 138 049 | 17.2 |
| **Maternal age at delivery, years** | |  |  |  |
| <25 | 3649 | 11.6 | 92 105 | 11.4 |
| 25-34 | 20 246 | 64.2 | 534 512 | 66.4 |
| 35-39 | 5886 | 18.7 | 143 485 | 17.8 |
| ≥40 | 1730 | 5.5 | 34 669 | 4.3 |
| Missing | 10 | <0.1 | 144 | <0.1 |
| **Parity** |  |  |  |  |
| 0 | 14 269 | 45.3 | 339 666 | 42.2 |
| 1-2 | 14 830 | 47.0 | 409 458 | 50.9 |
| ≥3 | 2338 | 7.4 | 51 904 | 6.4 |
| missing | 84 | 0.3 | 3887 | 0.5 |
| **Body Mass Index**** |  |  |  |  |
| Underweight | 829 | 2.6 | 18 841 | 2.3 |
| Normal | 14 894 | 47.3 | 423 426 | 52.6 |
| Overweight | 8103 | 25.7 | 200 403 | 24.9 |
| Obesity class I | 3821 | 12.1 | 77 123 | 9.6 |
| Obesity class II-III | 2048 | 6.5 | 33 986 | 4.2 |
| Missing | 1826 | 5.8 | 51 136 | 6.4 |
| **Maternal smoking, early pregnancy** | |  |  |  |
| Yes | 1556 | 4.9 | 32 610 | 4.1 |
| No | 27 992 | 88.8 | 719 895 | 89.4 |
| Missing | 1973 | 6.3 | 52 410 | 6.5 |
| **IVF pregnancy** |  |  |  |  |
| No | 29 845 | 94.7 | 715 140 | 88.8 |
| Yes | 1663 | 5.3 | 31 442 | 3.9 |
| Missing | 13 | <0.1 | 58 333 | 7.2 |
| **Gestational age at birth** |  |  |  |  |
| 22^+0^ to 23^+6^ | 69 | 0.2 | 295 | <0.1 |
| 24^+0^ to 27^+6^ | 330 | 1.0 | 1041 | 0.1 |
| 28^+0^ to 31^+6^ | 759 | 2.4 | 2498 | 0.3 |
| 32^+0^ to 36^+6^ | 3721 | 11.8 | 29 967 | 3.7 |
| 37^+0^ to 38^+6^ | 6946 | 22.0 | 163 527 | 20.3 |
| 39^+0^ to 40^+6^ | 11 248 | 35.7 | 430 629 | 53.5 |
| 41^+0^ to 41^+6^ | 7338 | 23.3 | 137 926 | 17.1 |
| ≥42^+0^ | 1110 | 3.5 | 39 032 | 4.8 |
| **Standardized birthweight***** | |  |  |  |
| <3^rd^ percentile | 7802 | 24.8 | 72 565 | 9.0 |
| 3^rd^ to 9.9^th^ percentile | 4126 | 13.1 | 93 398 | 11.6 |
| 10^th^ to 90^th^ percentile | 16 499 | 52.3 | 591 608 | 73.5 |
| 90.1^st^ to 97^th^ percentile | 1670 | 5.3 | 31 808 | 4.0 |
| >97^th^ percentile | 1424 | 4.5 | 15 233 | 1.9 |
| missing | 0 |  | 303 | <0.1 |
| **Sex** |  |  |  |  |
| Boy | 16 122 | 51.1 | 413 983 | 51.4 |
| Girl | 15 397 | 48.8 | 390 888 | 48.6 |
| Missing | 2 | <0.1 | 44 | <0.1 |

*Reference population includes all singleton live births in Sweden in 2014-2021, except cases with missing birthweight data (n=2712) or birthweight exceeding ± 5 SD (n=3792) according to Lindström et al^21^.

**Body mass index, classified according to the World Health Organization into underweight (<18.5 kg/m^2^), normal weight (18.5-24.9 kg/m^2^), overweight (25.0-29.9 kg/m^2^), obesity class I (30.0-34.9 kg/m^2^), and obesity class II-III (>35 kg/m^2^).

***Standardized birthweight according to Lindström et al^21^.

**Table S2 Univariable and adjusted risk ratios** **for poor fetal weight estimation (EFW more than ±10% from birth weight) by standardized estimated fetal weight, stratified by fetal sex**

|  | **UnivariableRR (95% CI)** | **Adjusted RR* (95% CI)** |
| --- | --- | --- |
| **Stratified on sex: females** |  |  |
| Severe SGA | **1.41 (1.32-1.51)** | **1.39 (1.30-1.48)** |
| Mild SGA | **1.11 (1.02-1.22)** | **1.12 (1.02-1.22)** |
| AGA | 1 (reference) | 1 (reference) |
| Mild LGA | 0.98 (0.84-1.15) | 0.99 (.84-1.16) |
| Severe LGA | **1.20 (1.04-1.38)** | **1.19 (1.03-1.38)** |
|  |  |  |
| **Stratified on sex: males** |  |  |
| Severe SGA | **1.54 (1.45-1.63)** | **1.49 (1.40-1.59)** |
| Mild SGA | **1.21 (1.12-1.32)** | **1.22 (1.12-1.32)** |
| AGA | 1 (reference) | 1 (reference) |
| Mild LGA | 0.85 (0.74-0.97) | 0.85 (0.74-0.98) |
| Severe LGA | 1.05 (0.92-1.19) | 1.04 (0.92-1.81) |

AGA, Appropriate for gestational age; CI, Confidence interval; LGA, Large for gestational age; SGA, Small for gestational age.

SGA, AGA, and LGA were classified as follows: severe SGA (<3rd percentile); mild SGA (3rd to <10th percentile); AGA (10th-90th percentile); mild LGA (>90th-97th percentile); and severe LGA (>97th percentile); all standardized for gestational age, and according to Lindström et al.

*Adjusted for gestational age at ultrasound (continuous variable) and for presentation at delivery (cephalic/non-cephalic)

**Table S3. Univariable and adjusted risk ratios for poor fetal weight estimation (EFW more than ±10% from birth weight) by standardized estimated fetal weight, stratified by fetal presentation.**

|  | **Univariable RR (95% CI)** | **Adjusted RR* (95% CI)** |
| --- | --- | --- |
| **Stratified on fetal presentation: cephalic** |  |  |
| Severe SGA | **1.42 (1.35-1.49)** | **1.41 (1.35-1.48)** |
| Mild SGA | **1.13 (1.06-1.20)** | **1.14 (1.07-1.21)** |
| AGA | 1 (reference) | 1 (reference) |
| Mild LGA | 0.89 (0.80-0.99) | 0.89 (0.79-0.99) |
| Severe LGA | 1.08 (0.97-1.19) | 1.06 (0.96-1.17) |
|  |  |  |
| **Stratified on fetal presentation: non-cephalic** |  |  |
| Severe SGA | **1.78 (1.55-2.03)** | **1.74 (1.52-2.00)** |
| Mild SGA | **1.49 (1.24-1.78)** | **1.51 (1.25-1.81)** |
| AGA | 1 (reference) | 1 (reference) |
| Mild LGA | 1.11 (0.76-1.62) | 1.10 (0.75-1.60) |
| Severe LGA | 1.54 (1.15-2.07) | 1.53 (1.14-2.06) |

AGA, Appropriate for gestational age; CI, Confidence interval; LGA, Large for gestational age; SGA, Small for gestational age.

SGA, AGA, and LGA were classified as follows: severe SGA (<3rd percentile); mild SGA (3rd to <10th percentile); AGA (10th-90th percentile); mild LGA (>90th-97th percentile); and severe LGA (>97th percentile); all standardized for gestational age, and according to Lindström et al.

*****Adjusted for gestational age at ultrasound (continuous variable) and for fetal sex (female/male)

**Table S4. Accuracy and precision of sonographic weight estimation stratified by fetal sex, by standardized estimated fetal weight, of pregnancies with known fetal sex (*n*=31,519).**

| **Fetal sex and standardized estimated fetal weight** | **Prevalence, *n* (%)** | **Mean percentage error* ± SD** | **P-value†** | **Estimated weight within ±10% of birthweight, *n*(%)** | **P-value‡** |
| --- | --- | --- | --- | --- | --- |
| **Female fetuses** |  |  |  |  |  |
| Severe SGA | 4581 (29.8) | -4.7 ± 9.7 | p<0.001 | 3185 (69.5) | p<0.001 |
| Mild SGA | 2376 (15.4) | -2.7 ± 8.4 |  | 1811 (76.2) |  |
| AGA | 7166 (46.5) | -1.1 ± 8.2 |  | 5694 (79.5) |  |
| Mild LGA | 646 (4.2) | 2.1 ± 7.2 |  | 531 (82.2) |  |
| Severe LGA | 628 (4.1) | 2.2 ± 7.9 |  | 491 (78.2) |  |
|  |  |  |  |  |  |
| **Male fetuses** |  |  |  |  |  |
| Severe SGA | 3798 (23.6) | -6.4 ± 9.8 | p<0.001 | 2428 (63.9) | p<0.001 |
| Mild SGA | 2164 (13.4) | -4.7 ± 8.7 |  | 1545 (71.4) |  |
| AGA | 8236 (51.1) | -2.5 ± 8.3 |  | 6336 (76.9) |  |
| Mild LGA | 1005 (6.2) | 1.3 ± 7.4 |  | 832 (82.8) |  |
| Severe LGA | 919 (5.7) | 1.2 ± 8.0 |  | 729 (79.3) |  |

AGA, Appropriate for gestational age; BMI, Body mass index; LGA, Large for gestational age; SD, standard deviation; SGA, Small for gestational age.

SGA, AGA, and LGA were classified as follows: severe SGA (<3rd percentile); mild SGA (3rd to <10th percentile); AGA (10th-90th percentile); mild LGA (>90th-97th percentile); and severe LGA (>97th percentile); all standardized for gestational age, according to Lindström et al.

*Mean percentage error (MPE), was calculated as the mean of the percentage errors ([EFW-BW]/EFW) ∙100

†One-way ANOVA was used to compare MPEs across the subgroups and post hoc testing using Bonferroni correction for multiple comparisons.

‡The Chi^2^ test was used for estimating the difference between groups in accuracy in weight estimation.

**Table S5. Accuracy and precision of sonographic weight estimation stratified by presentation at birth, by standardized estimated fetal weight at ultrasound, of pregnancies with known presentation at birth (*n*=30,450).**

| **Presentation at birth and standardized estimated fetal weight** | **Prevalence, *n* (%)** | **Mean percentage error* ± SD** | **P-value†** | **Estimated weight within ±10% of birthweight, *n*(%)** | **P-value‡** |
| --- | --- | --- | --- | --- | --- |
| **Fetuses with cephalic presentation at birth** | | | p<0.001 |  | p<0.001 |
| Severe SGA | 7438 (25.7) | -5.2 ± 9.6 |  | 5065 (68.1) |  |
| Mild SGA | 4180 (14.5) | -3.5 ± 8.5 |  | 3119 (74.6) |  |
| AGA | 14283 (49.4) | -1.8 ± 8.3 |  | 11,181 (78.3) |  |
| Mild LGA | 1562 (5.4) | 1.6 ± 7.2 |  | 1295 (82.9) |  |
| Severe LGA | 1448 (5.0) | 1.5 ± 7.9 |  | 1153 (79.6) |  |
|  |  |  |  |  |  |
| **Fetuses with non-cephalic presentation at birth** | | | p<0.001 |  | p<0.001 |
| Severe SGA | 943 (36.1) | -7.3 ± 11.2 |  | 549 (58.2) |  |
| Mild SGA | 360 (13.8) | -5.8 ± 9.0 |  | 237 (65.8) |  |
| AGA | 1119 (42.9) | -2.5 ± 8.6 |  | 849 (75.9) |  |
| Mild LGA | 89 (3.4) | 1.0 ± 9.1 |  | 68 (76.4) |  |
| Severe LGA | 99 (3.8) | 2.4 ± 9.3 |  | 67 (67.7) |  |

AGA, Appropriate for gestational age; BMI, Body mass index; LGA, Large for gestational age; SD, standard deviation; SGA, Small for gestational age.

SGA, AGA, and LGA were classified as follows: severe SGA (<3rd percentile); mild SGA (3rd to <10th percentile); AGA (10th-90th percentile); mild LGA (>90th-97th percentile); and severe LGA (>97th percentile); all standardized for gestational age, according to Lindström et al.

*Mean percentage error (MPE), was calculated as the mean of the percentage errors ([EFW-BW]/EFW) ∙100

†One-way ANOVA was used to compare MPEs across the subgroups and post hoc testing using Bonferroni correction for multiple comparisons.

‡The Chi^2^ test was used for estimating the difference between groups in accuracy in weight estimation.

**Table S6. Accuracy and precision of sonographic weight estimation stratified for gestational age, by standardized estimated fetal weight (*n*=31,521).**

| **Gestational age at ultrasound and standardized estimated fetal weight** | **Prevalence, *n* (%)** | **Mean percentage error* ± SD** | **P-value†** | **Estimated weight within ±10% of birthweight, *n* (%)** | **P-value‡** |
| --- | --- | --- | --- | --- | --- |
| **22+0-27+6** | | | 0.007 |  | 0.003 |
| Severe SGA | 203 (49.3) | -5.7 ± 12.6 |  | 115 (56.7) |  |
| Mild SGA | 33 (8.0) | -6.5 ± 10.1 |  | 23 (69.7) |  |
| AGA | 160 (38.8) | -2.8 ± 9.5 |  | 119 (74.4) |  |
| Mild LGA | 11 (2.7) | 1.0 ± 5.6 |  | 10 (90.9) |  |
| Severe LGA | 5 (1.2) | 6.2 ± 5.1 |  | 3 (60) |  |
| **28+0-31+6** | | | <0.001 |  | 0.002 |
| Severe SGA | 382 (48.4) | -4.5 ± 11.2 |  | 248 (64.9) |  |
| Mild SGA | 101 (12.8) | -3.9 ± 10.0 |  | 64 (63.4) |  |
| AGA | 282 (35.7) | -1.1 ± 9.1 |  | 210 (74.5) |  |
| Mild LGA | 11 (1.4) | 7.8 ± 10.3 |  | 3 (27.3) |  |
| Severe LGA | 13 (1.6) | 4.4 ± 9.9 |  | 10 (76.9) |  |
| **32+0-36+6** |  |  | <0.001 |  | <0.001 |
| Severe SGA | 1478 (36.3) | -5.3 ± 10.4 |  | 967 (65.4) |  |
| Mild SGA | 486 (11.9) | -4.9 ± 8.8 |  | 339 (69.8) |  |
| AGA | 1746 (42.9) | -3.3 ± 8.6 |  | 1284 (73.5) |  |
| Mild LGA | 107 (2.6) | -0.2 ± 9.4 |  | 73 (68.2) |  |
| Severe LGA | 256 (6.3) | -2.8 ± 9.5 |  | 176 (68.8) |  |
| **37+0-38+6** |  |  | <0.001 |  | <0.001 |
| Severe SGA | 1947 (26.3) | -5.3 ± 9.4 |  | 1317 (67.6) |  |
| Mild SGA | 931 (12.6) | -4.5 ± 8.7 |  | 665 (71.4) |  |
| AGA | 3376 (45.6) | -2.7 ± 8.3 |  | 2640 (78.2) |  |
| Mild LGA | 469 (6.3) | -0.6 ± 7.5 |  | 392 (83.6) |  |
| Severe LGA | 685 (9.2) | 1.7 ± 7.3 |  | 558 (81.5) |  |
| **39+0-40+6** |  |  | <0.001 |  | <0.001 |
| Severe SGA | 2756 (23.8) | -5.6 ± 9.3 |  | 1871 (67.9) |  |
| Mild SGA | 1745 (15.1) | -3.1 ± 8.3 |  | 1312 (75.2) |  |
| AGA | 6007 (51.9) | -1.6 ± 8.1 |  | 4725 (78.7) |  |
| Mild LGA | 640 (5.5) | 1.8 ± 6.8 |  | 550 (85.9) |  |
| Severe LGA | 420 (3.6) | 2.8 ± 7.3 |  | 341 (81.2) |  |
| **GA ≥ w 41+0** |  |  | <0.001 |  | <0.001 |
| Severe SGA | 1615 (22.2) | -5.6 ± 9.6 |  | 1096 (67.9) |  |
| Mild SGA | 1244 (17.1) | -3.3 ± 8.6 |  | 953 (76.6) |  |
| AGA | 3831 (52.7) | -0.8 ± 8.1 |  | 3052 (79.7) |  |
| Mild LGA | 413 (5.7) | 4.0 ± 6.3 |  | 335 (81.1) |  |
| Severe LGA | 168 (2.3) | 4.4 ± 7.2 |  | 132 (78.6) |  |

AGA, Appropriate for gestational age; BMI, Body mass index; LGA, Large for gestational age; SD, standard deviation; SGA, Small for gestational age.

SGA, AGA, and LGA were classified as follows: severe SGA (<3rd percentile); mild SGA (3rd to <10th percentile); AGA (10th-90th percentile); mild LGA (>90th-97th percentile); and severe LGA (>97th percentile); all standardized for gestational age, according to Lindström et al.

*Mean percentage error (MPE), was calculated as the mean of the percentage errors ([EFW-BW]/EFW) ∙100

†One-way ANOVA was used to compare MPEs across the subgroups and post hoc testing using Bonferroni correction for multiple comparisons.

‡The Chi^2^ test was used for estimating the difference between groups in accuracy in weight estimation.
